# Competitive kinetics versus stopped flow method for determining the degradation rate constants of steroids by ozonation

**DOI:** 10.1186/s40064-016-2782-4

**Published:** 2016-07-18

**Authors:** Alberto López-López, Valentín Flores-Payán, Elizabeth León-Becerril, Leonel Hernández-Mena, Ramiro Vallejo-Rodríguez

**Affiliations:** Department of Environmental Technology, Centro de Investigación y Asistencia en Tecnología y Diseño del Estado de Jalisco, A. C., Av. Normalistas 800, Colinas de la Normal, 44270 Guadalajara, Jalisco Mexico

**Keywords:** Steroids, Competitive kinetics, Stopped flow, Second order constant

## Abstract

Steroids are classified as endocrine disrupting chemicals; they are persistent with low biodegradability and are hardly degraded by conventional methods. Ozonation process has been effective for steroids degradation and the determination of the kinetics is a fundamental aspect for the design and operation of the reactor. This study assessed two methods: competitive kinetics and stopped flow, for determining the degradation kinetics of two steroids, estradiol (E2) and ethinylestradiol (EE2) in spiked water. Experiments were performed at pH 6, 21 °C, and using tertbutyl alcohol as scavenger of hydroxyl radicals; competitive kinetics method used sodium phenolate as reference compound. For the stopped flow, the experiments were performed in a BioLogic SFM-3000/S equipment. For both methods, the second order rate constants were in the order of 10^6^ and 10^5^ M^−1^ s^−1^ for E2 and EE2 respectively. The competitive kinetics can be applied with assurance and reliability but needing an additional analysis method to measure the residual concentrations. Stopped flow method allows the evaluation of the degradation kinetics in milliseconds and avoids the use of additional analytical methodologies; this method allows determining the reaction times on line. The methods are applicable for degradation of other emerging contaminants or other steroids and could be applied in water treatment at industrial level. Finally, it is important to consider the resources available to implement the most appropriate method, either competitive kinetics or the stopped-flow method.

## Background

Emerging contaminants are chemical compounds comprising products used in anthropogenic activities, including human and veterinary pharmaceuticals, personal care products, surfactants, pesticides, plasticizers, drugs and industrial additives (Snyder et al. 2006). In particular, endocrine-disrupting chemicals (EDCs) are emerging contaminants which are persistent with low biodegradability and mainly interfere with the endocrine systems of various species (Gomes et al. 2011; Burkhardt-Holm et al. 2008; Peng et al. 2008; Suárez et al. 2008). Conventional treatment processes are not efficient for the removal of EDCs in water (Gomes et al. 2011), thus these contaminants have been detected at different concentration levels of ng L^−1^ and μg L^−1^ in wastewater with or without treatment (Samaras et al. 2010; Santos et al. 2005), and in surface and groundwater (Benotti et al. 2009, 2010). In surface water such as rivers and lakes, which are considered a source of drinking water, has been detected the presence of EDCs, in particular pharmaceuticals including steroids (Huerta-Fontela et al. 2011; Alvarez et al. 2008; Benotti et al. 2009; Gibson et al. 2007), implying a potential public health problem (von der Ohe et al. 2011).

Ozonation process has been useful for the degradation of steroids in water reaching removal efficiencies up to 80 % (Huber et al. 2003, 2005; Deborde et al. 2005); it has been applied at pilot scale for the treatment of effluents from a wastewater treatment plant containing pharmaceutical drugs and steroids including estradiol and ethinylestradiol (Huber et al. 2005). One important aspect in the design of reactors in ozonation process is the knowledge of the kinetics constants; in particular, the reaction between ozone and steroids follows a second order rate ranging from 10^4^ to 10^5^ L mol^−1^ s^−1^, thus the reaction is very fast and the methodology for achieving the constants should have high precision and be reliable to asses constants for this order of magnitude. The second order rate constant (${\text{k}}_{{{\text{O}}_{ 3} , {\text{M}}}}$) can be determined by competitive kinetics (Deborde et al. 2005; Gurol and Nekouinaini 1984; Hoigné and Bader 1983) and stopped flow and quenched flow methods (Chelme-Ayala et al. 2010; Huber et al. 2005; Ledakowicz et al. 2001).

Competitive kinetics method consists of the simultaneous degradation of two compounds; one of them is the reference compound whose rate constant is known and the other one is the compound whose constant is unknown; both compounds must have the same order of reaction with respect to ozone (Benitez et al. 2009; Huber et al. 2003). Stopped flow method is a spectroscopic technique, which involves the injections of solutions into a mixing chamber and wherein after a very short time, ms, the flow is suddenly stopped. The mixing chamber is irradiated with a monochromatic light, measuring the change signal of the reaction with respect to time, generally by absorbance (Chelme-Ayala et al. 2010). These methodologies have particular characteristics, competitive kinetics depends on the kinetic rate constant of the reference compound and implies additional analysis to determine the residual concentration of compounds; while the stopped flow technique depends on the amperage stability in the equipment to obtain the screen signal and on the relatively easy in which the oxidant and the steroid are spectrophotometrically monitored.

Both methodologies have been performed to determine the kinetics of steroids degradation in water by ozonation. Huber et al. (2003) degraded estradiol and ethinylestradiol by ozonation, obtaining second order rate constants by competitive kinetics in the order of 10^4^ and 10^5^ L mol^−1^ s^−1^, and establishing that kinetics is strong dependent to changes in pH and ozone doses. Deborde et al. (2005) degraded steroids with ozone doses of 4 × 10^−8^ mol L^−1^, achieving a removal efficiency of 95 %, and obtaining a second order constant between 10^4^ and 10^5^ L mol^−1^ s^−1^ by competitive kinetics. Also, by competitive kinetics, Vallejo-Rodríguez et al. (2014) determined the kinetics constant in the order of 10^5^–10^6^ L mol^−1^ s^−1^ for 17β-estradiol and 17α-ethinylestradiol in water, emphasizing the importance of the stoichiometric coefficients of the main reaction, which are others factors to consider in the design and scaling up of the process. In contrast, the stopped-flow method is scarcely reported in steroid degradation with ozone. Adams et al. (2005) degraded E2 and EE2 by ozonation to analyze the estrogenicity of degradation products.

The aim of this research is to compare two methods, competitive kinetics versus stopped flow, in the determination of second-order rate constants of EDCs in spiked water samples by ozonation, taking as model compounds two steroids, 17β-estradiol and 17α-ethinylestradiol. The assessment of technical advantages, disadvantages and the importance of the necessary scientific infrastructure of the two methods are highlighted.

## Methods

### Standards and reagents

As EDCs, two steroids 17β-estradiol (E2) and 17α-ethinylestradiol (EE2) were chosen as target compounds, because they are frequently identified in the environment as a consequence of their high public consumption (Vajda et al. 2008; Fent et al. 2006). E2 is a natural hormone, relatively bioaccumulative and persistent in the environment while EE2 is a synthetic hormone from cholesterol and the active ingredient in birth control pills (Maniero et al. 2008).

Standards for E2 (98 %) and EE2 (98 %) were acquired in powder form from Sigma–Aldrich and Fluka (USA). As reference compound in competitive kinetics, sodium phenolate 99 % was used; potassium dihydrogen phosphate (KH_2_PO_4_) and tert-butyl alcohol were acquired from Sigma–Aldrich (USA). Tert-butyl alcohol was used as scavenger of OH radical in the experiments (reagent grade) and without further purification. All solvents were HPLC grade; acetonitrile was acquired from Tedia (USA), methanol from JT Baker (USA) and ethyl acetate from Burdick and Jackson (USA). Derivatization was performed directly using N,O-bis (trimethylsilyl) trifluoroacetamide (BSTFA) + 1 % trimethylchlorosilane (TMCS), from Sigma-Aldrich. Stock solutions of the selected compounds were prepared with deionized water (Millipore). Ozone gas was generated from a Pacific Ozone G11 equipment operated at 20 °C from pure O_2_, and then MilliQ water was saturated with ozone gas to obtain the standard solution of ozone (0.25 mmol L^−1^).

### Analytical methods

Steroids concentration was determined by solid phase extraction; sodium phenolate concentration in the extracted samples was determined by high performance liquid chromatography (HPLC) using a Varian ProStar 7725 equipment with a Varian ProStar 230 diode array detector (DAD) (Walnut Creek, CA) using maximal absorption wavelengths (λ_max_) of 197 nm following the methodology from Vallejo-Rodríguez et al. (2011). Residual steroid was determined by gas chromatography (GC) using an Agilent Technologies chromatograph model 6890 coupled with a mass spectrometer (MS) 5975 and with a quadruple mass filter with an autosampler model 7683, prior the sample was derivatized. The GC was equipped with a HP5MS 30 m × 0.25 mm capillary column (Agilent, USA), 0.25 mm internal diameter (i.d.) with a stationary phase of 5 % phenyl and 95 % dimethyl polysiloxane, and a 0.25 μm film thickness. Oven temperature was programmed at 120 °C for 20 min, ramped at 15 °C min^−1^ to 250 °C and finally increased 5 °C min^−1^ up to 300 °C and held for 5 min. The injector temperature was 300 °C in splitless mode using an injection volume of 1.0 μL. Helium (99.999 %, INFRA) was used as the carrier gas at a constant flow rate of 1.0 mL min^−1^. Mass spectra was obtained by electron impact (EI) at 70 eV using ionization source at 200 °C. Mass scanning was used in SCAN mode for optimizing the separation and identification of compounds and selected ion monitoring (SIM) for quantification (Bowden et al. 2009). Ozone concentration in water was performed by indigo colorimetric method 4500 (Eaton 2005).

### Determination of rate constants

#### Competitive kinetics method

Competitive kinetics was performed by duplicate in batch reactors using 50 mL volumetric flasks at T = 20 ± 1 °C and pH = 6 to ensure the molecular action of ozone and prevent fast decomposition into hydroxyl radicals. Steroid/sodium phenolate ratio was equimolar; E2/ozone and EE2/ozone stoichiometric ratios were 5:1 and 1:5 from standard solutions of 3.6 and 11.8 μmol L^−1^ for E2 and EE2, respectively (Huber et al. 2003). In general form, the model for the competitive kinetics method is expressed by Eq. (1) (Benitez et al. 2009; Huber et al. 2003).

1 $$ { \ln }\left( {{{\left[ {\text{M}} \right]_{ 0} } \mathord{\left/ {\vphantom {{\left[ {\text{M}} \right]_{ 0} } {\left[ {\text{M}} \right]_{\text{t}} }}} \right. \kern-0pt} {\left[ {\text{M}} \right]_{\text{t}} }}} \right)\,\, = \,\,\frac{{{\text{n}}_{\text{M}} }}{{{\text{n}}_{\text{Phen}} }}\left( {\frac{{{\text{k}}_{{{\text{O}}_{ 3} , {\text{M}}}} }}{{{\text{k}}_{{{\text{O}}_{ 3} , {\text{Phen}}}} }}} \right)\,{ \ln }\,\left( {{{\left[ {\text{Phen}} \right]_{ 0} } \mathord{\left/ {\vphantom {{\left[ {\text{Phen}} \right]_{ 0} } {\left[ {\text{Phen}} \right]_{\text{t}} }}} \right. \kern-0pt} {\left[ {\text{Phen}} \right]_{\text{t}} }}} \right) $$

Here [M]_0_ and [M]_t_ are the concentrations of the steroid at t = 0 and at a time t, respectively, n_M_ is the stoichiometric coefficient and ${\text{k}}_{{{\text{O}}_{ 3} , {\text{M}}}}$ is the second order rate constant. Sodium phenolate was used as the reference compound, thus $\left[ {\text{Phen}} \right]$, ${\text{n}}_{\text{Phen}}$ and ${\text{k}}_{{{\text{O}}_{ 3} , {\text{Phen}}}}$ represent the concentration, the stoichiometric coefficient and the rate constant of sodium phenolate, respectively. Stoichiometric coefficients for E2 and EE2 used in this work was taken from Vallejo-Rodríguez et al. (2014), where 1 mol of ozone is necessary to oxidize 1 mol of E2 or EE2.

From Eq. (1), the plot ${ \ln }\left( {{{\left[ {\text{M}} \right]_{ 0} } \mathord{\left/ {\vphantom {{\left[ {\text{M}} \right]_{ 0} } {\left[ {\text{M}} \right]_{\text{t}} }}} \right. \kern-0pt} {\left[ {\text{M}} \right]_{\text{t}} }}} \right)$ versus ${ \ln }\left( {{{\left[ {\text{Phen}} \right]_{ 0} } \mathord{\left/ {\vphantom {{\left[ {\text{Phen}} \right]_{ 0} } {\left[ {\text{Phen}} \right]_{\text{t}} }}} \right. \kern-0pt} {\left[ {\text{Phen}} \right]_{\text{t}} }}} \right)$, gave the slope ${{{\text{k}}_{{{\text{O}}_{ 3} , {\text{M}}}} } \mathord{\left/ {\vphantom {{{\text{k}}_{{{\text{O}}_{ 3} , {\text{M}}}} } {{\text{k}}_{{{\text{O}}_{ 3} , {\text{Phen}}}} }}} \right. \kern-0pt} {{\text{k}}_{{{\text{O}}_{ 3} , {\text{Phen}}}} }}$, thus ${\text{k}}_{{{\text{O}}_{ 3} , {\text{Phen}}}}$ is known, and ${\text{k}}_{{{\text{O}}_{ 3} , {\text{M}}}}$ can be determined, here ${\text{k}}_{{{\text{O}}_{ 3} , {\text{Phen}}}}$ = 2.4 × 10^5^ L mol^−1^ s^−1^ at pH 6 (Hoigné and Bader 1983).

#### Stopped flow method

The acquisition of kinetic data and photometric measurements by stopped flow were performed on a BioLogic SFM-3000/S equipment. Stoichiometric ratios from 1:1 to 1:20 for E2/ozone and EE2/ozone were established injecting with the syringe system of the equipment (corresponding to 1:1 to 1:5 for E2-EE2/ozone volume ratios), different volumes of ozone at pH = 6 to ensure the molecular action of ozone and prevent fast decomposition of ozone into hydroxyl radicals. For both steroids, the detections were performed at 197 nm. In order to obtain the second order kinetic constant, the first step is the determination of the absolute rate constant under pseudo-first-order conditions (Eq. (2)):

2 $$ { \ln }\left( {{{\left[ {\text{M}} \right]_{ 0} } \mathord{\left/ {\vphantom {{\left[ {\text{M}} \right]_{ 0} } {\left[ {\text{M}} \right]_{\text{t}} }}} \right. \kern-0pt} {\left[ {\text{M}} \right]_{\text{t}} }}} \right) = {\text{k}}_{\text{obs}} \,{\text{t}} $$

For each experiment performed in the stopped flow, a value of k_obs_ is obtained by plotting ${ \ln }\left( {{{\left[ {\text{M}} \right]_{ 0} } \mathord{\left/ {\vphantom {{\left[ {\text{M}} \right]_{ 0} } {\left[ {\text{M}} \right]_{\text{t}} }}} \right. \kern-0pt} {\left[ {\text{M}} \right]_{\text{t}} }}} \right)$ versus time, where the slope is k_obs_. Here k_obs_ is the pseudo-first-order rate constant and t is time.

From Eq. (3), ${\text{k}}_{{{\text{O}}_{ 3} }}$ values were obtained from the slope by plotting values of k_1_ versus $[ {\text{O}}_{ 3} ]_{ 0}$ (Hoigné et al. 1985):

3 $$ {\text{k}}_{ 1}  = {\text{k}}_{{{\text{O}}_{ 3} ,{\text{M}}}} [ {\text{O}}_{ 3} ]_{ 0} $$

Here ${\text{k}}_{{{\text{O}}_{ 3} }}$ is the second order rate constant for M and $[ {\text{O}}_{ 3} ]_{ 0}$ is the initial concentration of ozone for each experiment.

## Results and discussion

### Competitive kinetics method

**Fig. 1:**
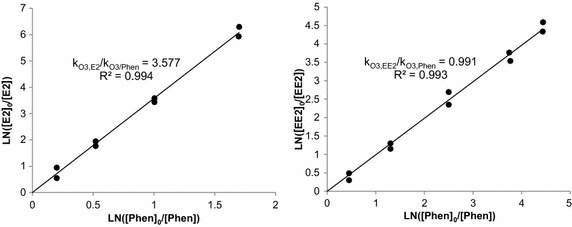
Rate kinetic using competition kinetics at pH 6, t = 20 °C. **a** Obtaining ${\text{k}}_{{{\text{O}_{3}},\text{E2}}}$ for [E2]_0_ = 0.18 μmol L^−1^, **b** Obtaining ${\text{k}}_{{{\text{O}_{3}},\text{EE2}}}$ for [EE2]_0_ = 0.59 μmol L^−1^

From Eq. (1) and Fig. 1a, b, the slopes of ${\text{k}}_{{{\text{O}}_{ 3} , {\text{M}}}} / {\text{k}}_{{{\text{O}}_{ 3} , {\text{Phen}}}}$ were determined for each steroid. From Fig. 1a, ${\text{k}}_{{{\text{O}}_{ 3} , {\text{E2}}}} / {\text{k}}_{{{\text{O}}_{ 3} , {\text{Phen}}}}$ is 3.577; then ${\text{k}}_{{{\text{O}}_{ 3} , {\text{E2}}}}$ = 0.73 × 10^6^ L mol^−1^ s^−1^ for E2 was obtained at pH 6 and 20 °C, with a multiple determination coefficient R^2^ = 0.994 and a variation coefficient CV < 10.0 %. For E2, from Fig. 1b, ${\text{k}}_{{{\text{O}}_{ 3} , {\text{EE2}}}} / {\text{k}}_{{{\text{O}}_{ 3} , {\text{Phen}}}}$ = 0.991, then ${\text{k}}_{{{\text{O}}_{ 3} , {\text{EE2}}}}$ = 2.040 × 10^6^ L mol^−1^ s^−1^ with R^2^ = 0.993 and CV < 5.0 %, at pH 6 and 20 °C.

Degradation efficiencies for both steroids, E2 and EE2, were above 99 % for the assayed ozone doses. Degradation time can be estimated (Hoigné et al. 1985) it ranged from 3.5 to 90 s and from 8 to 160 s for E2 and EE2, respectively; degradation time decreases if ozone dose increases (data no shown). For the smallest ozone dose applied, 0.05 μmol L^−1^, the half-life time (t_1/2_) was lesser than 16 s for E2 and 29 s for EE2; while for the greater ozone dose, 1.5 μmol L^−1^, t_1/2_ was 0.6 s for E2 and 0.90 s for EE2, these values are in accordance to that reported by Huber et al. (2003) and Vallejo-Rodríguez et al. (2014).

### Stopped flow method

**Fig. 2:**
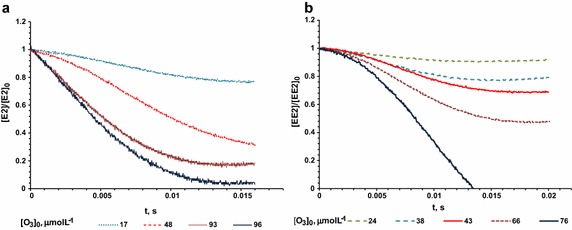
Degradation kinetics of pseudo-first-order of steroids by stopped flow method using stoichiometric ratios from 1:1 to 1:20 for E2/ozone (**a**) and for EE2/ozone (**b**) from the injection of different volumes of ozone

**Fig. 3:**
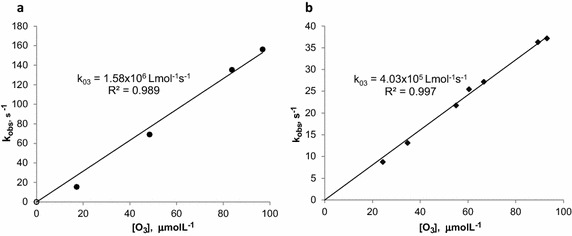
Obtaining of second order constant by stopped flow kinetics for different ozone doses: **a** E2 (${\text{k}}_{{{\text{O}}_{ 3} , {\text{E2}}}}$ = 1.58 × 10^6^ L mol^−1^ s^−1^) and **b** EE2 (${\text{k}}_{{{\text{O}}_{ 3} , {\text{EE2}}}}$ = 4.03 × 10^5^ L mol^−1^ s^−1^)

Figure 2a, b show the experimental results from the degradation kinetics of E2 and EE2, respectively performed by the stopped flow method. For each experiment at different ozone concentration, k_obs_ was obtained by Eq. (2) and then ${\text{k}}_{{{\text{O}}_{ 3} , {\text{M}}}}$ from Eq. (3), obtaining 1.58 × 10^6^ and 4.03 × 10^5^ L mol^−1^ s^−1^ for E2 and EE2, respectively (see Fig. 3a, b). Degradation efficiencies of E2 and EE2 for the highest ozone doses (96 mmol L^−1^ for E2 and 76 mmol L^−1^ for EE2) were above 96 and 99 %, respectively, which can be observed in Fig. 2. Degradation time was obtained following Hoigné et al. (1985), it is in the order of milliseconds and <0.02 s; the t_1/2_ was <10 ms for E2 and 14 ms for EE2 (Fig. 2a, b).

## Discussion

Table 1 resumes the values of kinetic constants for E2 and EE2 obtained in this study and that reported in the literature at 20 °C and pH = 6. The order of magnitude of the kinetic constants by competitive kinetics and stopped flow method is 10^6^ and 10^5^ L mol^−1^ s^−1^ for E2 and EE2, respectively. In this study, ${\text{k}}_{{{\text{O}}_{ 3} , {\text{M}}}}$ values obtained by competitive kinetics are very similar to that determined by Deborde et al. (2005), Vallejo-Rodríguez et al. (2014) and Huber et al. (2003) for E2, using the same compound of reference and a similar liquid chromatography methodology to quantify the residual concentration of E2. Slight differences for ${\text{k}}_{{{\text{O}}_{ 3} , {\text{M}}}}$ by competitive kinetics are found which can be explained to the presence of organic matter in natural water (Huber et al. 2003) and which can consume as much as ozone. Also, the values in the stoichiometric coefficients in Eq. (1) can affect the ${\text{k}}_{{{\text{O}}_{ 3} , {\text{M}}}}$ values (research herein and Vallejo-Rodríguez et al. 2014). These differences are more evident for EE2, in which the kinetics constants in this study are greater than that obtained by Deborde et al. (2005) and Vallejo-Rodríguez et al. (2014).

**Table 1:** Comparison of kinetic rate constants of second order in ultrapure water by competitive kinetic and stopped flow

Compound	μmol L^−1^	pH, T (°C)	Method of evaluation	Aqueous matrix	Compound of reference	${\text{k}}_{{{\text{O}}_{ 3} , {\text{M}}}}$ (L mol^−1^ s^−1^)
E2	1.8	6, 20	Competitive kinetics	Spiked water	Phenolate	0.73 × 10^6, ^*
	3.6	6, 20	Stopped flow	Spiked water	nr	1.58 × 10^6,^ *
	1	6, 20	Competitive kinetics	Spiked water	Phenol	0.37 × 10^6, a^
	0.80	6, 20	Competitive kinetics	Spiked water	Phenolate	0.90 × 10^6, b^
	0.15	6, 20	Absolute rate constant	Spiked water	nr	0.90 × 10^6, c^
	0.28	6, 20	Absolute rate constant	Natural water	nr	0.99 × 10^6, c^
	4	6, 20	Competitive kinetics	Natural water	Phenolate	~1.00 × 10^6, d^
EE2	5.9	6, 20	Competitive kinetics	Spiked water	Phenolate	2.04 × 10^5,^ *
	11.8	6, 20	Stopped flow	Spiked water	nr	4.00 × 10^5,^ *
	1	6, 20	Competitive kinetics	Spiked water	Phenol	1.83 × 10^5, a^
	6.40	6, 20	Competitive kinetics	Spiked water	Phenolate	0.73 × 10^5, b^
	0.14	6, 20	Absolute rate constant	Spiked water	nr	0.73 × 10^6, c^
	0.25	6, 20	Absolute rate constant	Natural water	nr	3.16 × 10^5, c^
	4	6, 20	Competitive kinetics	Natural water	Phenolate	3.16 × 10^5, d^

* Research herein; nr: not required

^a^Deborde et al. (2005)

^b^Vallejo-Rodríguez et al. (2014)

^c^Vallejo-Rodríguez et al. (2015)

^d^Huber et al. (2003)

Values of ${\text{k}}_{{{\text{O}}_{ 3} , {\text{M}}}}$ obtained by stopped flow are twice the values obtained by competitive kinetics for both steroids; this can be due to the fate that competitive kinetics is subject to a reference compound, in this study sodium phenolate, which competes with the steroid for consumption of ozone. However, in the stopped flow equipment, only the steroids react with ozone. In addition, competitive kinetics is subject to the effectiveness of analytical methods to determine the residual steroids. In contrast, with the stopped flow method, the concentration of steroids is directly determined from the equipment at the same time the reaction occurs. Another difference among the methods is that reaction time in competitive kinetics is calculated, however in the stopped flow, it is displayed in real time and only depends on the concentrations of the residual steroid. Thus, the advantage of using the stopped flow methodology is the certitude in the generation of absorbance data at the same time while the reaction happens.

The reaction times of both competitive kinetics and stopped flow methods are consistent with the ozone dose applied in the experiments. Half-life time of steroids for the competitive kinetics was 1600 times greater than that determined by the stopped flow method. The ozone doses used by the competitive kinetics were 64 times lesser than those used by the stopped flow method. This deduction indicates that half-time is inversely proportional to the ozone doses in both methods. Hence, the high doses of ozone applied prove the obtaining of short times of half-life for both steroids by the stopped flow method.

## Conclusions

Competitive kinetics and stopped flow methods are reliable, fast and useful for determining the kinetics of steroids degradation in water by ozonation, and in general to evaluate fast degradation kinetics between emerging contaminants and ozone. Second order constants determined by both methods were similar to the results reported by the literature. Both methods have different technical advantages and require different analytical equipment for their application. The competitive kinetic can be applied with assurance and reliability but it needs further analysis methods to determine the residual concentrations of organic compounds involved in the reaction system and consequently a MS-GC. The method stopped flow evaluates the kinetics of degradation in real time (in milliseconds order) also optimizes the use of chemical reagents and the use of analytical methods to quantify residual compound is optional, then the evaluation in time of kinetics degradation is faster compared to the method of competitive kinetics, however this method needs a stopped-flow equipment.
